# The role of household composition in social isolation and anxiety: evidence from adults in South Africa during the COVID-19 pandemic

**DOI:** 10.1093/geronb/gbag076

**Published:** 2026-04-26

**Authors:** Elyse A Jennings, Carla Roberts-Toler, Nigel W Harriman, Daniel Ohene-Kwofie

**Affiliations:** Harvard Center for Population and Development Studies, Harvard T.H. Chan School of Public Health, Cambridge, Massachusetts, United States; Harvard Center for Population and Development Studies, Harvard T.H. Chan School of Public Health, Cambridge, Massachusetts, United States; Department of Social and Behavioral Sciences, Harvard T.H. Chan School of Public Health, Boston, Massachusetts, United States; MRC/Wits Rural Public Health and Health Transitions Research Unit (Agincourt), School of Public Health, Faculty of Health Sciences, University of the Witwatersrand, Johannesburg, South Africa; (Social Sciences Section)

**Keywords:** Caregiving, COVID-19, Social isolation, Living arrangements

## Abstract

**Objectives:**

Social isolation is a concern among aging populations, and household composition may play an important role in its impact on mental health. We use data from an aging cohort in rural South Africa to investigate the moderating role of household size and both gender and age composition in how declining extra-household social contact during the COVID-19 pandemic impacted anxiety.

**Methods:**

The participants included in the data completed both wave 3 of Health and Aging in Africa: A Longitudinal Study of an INDEPTH Community (HAALSI) and a temporally overlapping COVID-19 phone survey (July 2021–March 2022) (*n *= 2,054). We measured anxiety using a modified 2-item Generalized Anxiety Disorder scale and used household size and the numbers of women, men, older adults (ages 60+), and children (<age 15) in the household as modifying measures. Analyses used gender-stratified generalized estimating equations with a logit link.

**Results:**

Among both women and men, a decrease in in-person social interaction was associated with greater odds of increased anxiety during the same time period. Living in larger households, households with more men, and households with more children increased the magnitude of the association between decline in in-person interactions and odds of increased anxiety for women. We did not find significant modifying effects for men.

**Discussion:**

We speculate that these results stem largely from the caregiving burden, which falls more heavily on women than men, especially among the aging population in this setting. Furthermore, they have implications for aging adults who may experience greater social isolation with age.

As populations age, social isolation is a growing concern (Courtin & Knapp, 2017). The most rapidly aging populations are in low- and middle-income countries (LMICs), where multiple generations or extended families often reside in the same household (Bongaarts, 2001). While more time spent at home in older age can sometimes be a risk factor for social isolation (Toepoel, 2013), those who are aging within extended-family households may face lower, or at least different, risks. Co-resident family members may offer protection against the negative consequences that extra-household social isolation can have for mental health, but there is also potential for these large and mixed-generation households to exacerbate the negative consequences. This paper investigates the role of household size and composition in anxiety outcomes among an aging cohort in rural South Africa, during a time of heightened social isolation: the early months of the COVID-19 pandemic. Embedded within a pre-existing longitudinal study, data collected during the pandemic offer the unique opportunity to identify whether certain aspects of household composition might be protective (or harmful) during a period when individuals were isolating from extra-household social ties.

Few studies of the impacts of physical distancing on older adults during the COVID-19 pandemic have focused on sub-Saharan African (SSA) populations, despite the unique challenges faced by aging adults in these settings. In SSA, high prevalence of HIV-related mortality has hollowed out the middle generation, often leaving older adults as the primary caregivers for their grandchildren (Schatz & Seeley, 2015). The gendered impacts of this hallowing are pervasive, with women most often assuming the role of caregiver (Schatz & Seeley, 2015), similar to other settings (Bird, 1999; Verbakel et al., 2017). In SSA settings, social norms related to masculinity tend to be particularly strong, and these norms conflict with the caregiver role (Schatz & Seeley, 2015). These pre-existing family and gender dynamics suggest that physical distancing may have uniquely detrimental impacts on older adults, particularly women, in SSA. By examining the impacts of physical distancing during the COVID-19 pandemic in an SSA setting, the present study advances knowledge of associations between social isolation, resulting from public health-mandated physical distancing, and mental health across settings.

In this paper, we investigate the association between self-reported physical distancing during the first year and a half of the COVID-19 pandemic and self-reported increase in anxiety symptoms during the same timeframe. We use data from Health and Aging in Africa: Longitudinal Studies in South Africa INDEPTH (HAALSI), a study of aging Black South African adults in rural Agincourt, who were aged 40+ at the baseline wave, in 2014–2015. Many studies that have focused on mental health during the COVID-19 pandemic use data collected in the first few months following initial lockdowns (Choi et al., 2022; Dagnino et al., 2020; Etheridge & Spantig, 2020; Kim & Jung, 2021; Marroquín et al., 2020; Okabe-Miyamoto et al., 2021; Pancani et al., 2021). The data used in this investigation were collected beginning in July 2021, approximately 16 months after the initial lockdowns were put into place in South Africa, allowing us to investigate the longer-term impacts of physical distancing. The key measures we use come from survey questions asking respondents’ self-perceived increase/decrease/no change in both the frequency with which they have in-person interactions and their anxiety since the start of lockdowns (March 2020). We identify the extent to which the composition of people’s households may have either buffered or exacerbated the association between these measures of physical distancing and anxiety. We look beyond simply household size to understand the likely important role that the gender and age composition of the household played in either buffering against or exacerbating the negative impacts of physical distancing on anxiety.

## Conceptual framework

### Background and setting

The Agincourt study area, located in the northeast of South Africa, is a rural, low-income sub-district where Black South Africans belonging to the Shangaan ethnic group were forcibly moved during Apartheid (Blalock, 2014). Employment opportunities are limited, and poverty rates are high (Riumallo Herl et al., 2022). Because of a lack of employment opportunities as well as the legacy of Apartheid policies, circulatory labor migration is common. Migration is most common among young men, and more common among young women now than in the past, but is not very common among the older population (Collinson et al., 2014).

South Africa faces a high burden of disease, with HIV impacting health and mortality at relatively young ages (Mee et al., 2016). High rates of mortality have put South Africa and Agincourt at a large disadvantage in life expectancy, when compared with other world regions. Life expectancy at birth in Agincourt was estimated to be 74 years for women and 67.7 years for men in 2018, which was a few years higher than estimates for the nationwide population (Kabudula et al., 2021), but still 14 to 16 years lower than in North America and Europe the same year (Population Reference Bureau, 2018).

In Agincourt, families and households tend to be large. Total fertility is now close to replacement level in Agincourt, but women were still having more than four children, on average, as recently as the mid-1990s (Williams et al., 2013). Households are often multigenerational, and multiple relatives may co-reside in a small space (Wittenberg & Collinson, 2007). In Agincourt, the average household size is five to six members, compared with less than three members in U.S. households (Mather et al., 2019). As a result of Apartheid-era policies that compelled men to leave their families to accept jobs in the outskirts of cities (Hosegood et al., 2009), it is not uncommon for households to be headed by women. About 40% of households in Agincourt are female-headed (Schatz et al., 2011). Women are more likely to head extended family households than nuclear family households (Dungumaro, 2008).

South Africa had one of the highest excess death rates when COVID-19 first broke out, with an excess of over 42,000 deaths recorded between January and August 2020 (Bradshaw et al., 2021; Dorrington et al., 2022; Harling et al., 2021). Stringent lockdowns were imposed nationwide, beginning on March 23, 2020 (Bhorat et al., 2021), as part of measures to control the spread of the virus. Lockdowns resulted in school closures and limitations to travel and non-essential activities (Harriman et al., 2024). Because of the restrictions on working outside the home, in April 2020, the government allocated R50 billion (approximately $3.04 billion) to social assistance for the most vulnerable households. In addition to increasing existing social grants, this money also funded a new COVID-19 Social Relief of Distress grant, which provides temporary (three-month) assistance in the form of R350 (approximately $21) per month to individuals not receiving other social grants (Bhorat et al., 2021). This was about 10% of the monthly minimum wage at the time, assuming a 40-hour work week at the hourly minimum wage of R20.76 (Ngundu, 2025). These grants may have helped to offset some of the negative impacts of physical distancing.

### The importance of household composition for mental health during the pandemic

Across the globe, the COVID-19 pandemic intensified the already-existing risk of social isolation among aging adults (Choi et al., 2022; Dury, 2014; Emerson, 2020). In the absence of knowledge about the COVID-19 virus and how to prevent its spread, physical distancing was the most available and effective tool. Stay-at-home orders were announced around the world, advising people to physically distance from others and only leave their homes when necessary. Older adults were especially likely to physically distance themselves from others, or to have loved ones distance themselves from them, given the early evidence that older people suffered worse symptoms and heightened risk of mortality if they contracted the virus (Hwang et al., 2020; Yanez et al., 2020). Physical distancing has been found, across settings, to be linked to lower mental health, including depression, loneliness, and anxiety (Benke et al., 2020; Chen et al., 2021; Wei, 2020).

As people isolated themselves from the outside world, they spent most or all of their time at home, increasing the salience of coresident household members to their daily lives and well-being. Coresident household members have the potential to increase or decrease the quality of one’s life and mental health. This can depend on relationship dynamics between focal individuals and their fellow household members, as well as the contributions that fellow household members make to the household (Rao et al., 2020; Zhang, 2022). One study showed that living with others (vs alone) was associated with worse mental health symptoms among a small U.S.-based sample (Wei, 2020), and another study across 151 countries found that living in larger households was associated with increased anxiety among individuals staying home at the beginning of the pandemic (Paudel, 2021). There is also evidence that *who* a person lived with, not just how many people they lived with, during the early stages of the pandemic was important for their social connection and mental health (McDonald et al., 2022; Okabe-Miyamoto et al., 2021).

### Expectations for impact of household composition on anxiety in rural South Africa

#### Household size

In South Africa, multigenerational and large households are common (Abrahams et al., 2022; Amoateng et al., 2007). Living in larger households provided a greater number of people with whom an individual may interact regularly while physical distancing measures were in place. These social interactions may have buffered against feelings of isolation and loneliness and, in turn, resulted in less anxiety (Kung et al., 2022; Okruszek et al., 2020). Living with more people may also have allowed for the sharing of financial or caregiving responsibilities, which could help alleviate individuals’ stress and anxiety.

On the other hand, individuals living in larger households during the pandemic may have felt more anxious for at least a few possible reasons. First, larger households may have increased the potential for conflict between any two or more household members, creating or exacerbating stress and tension for all members (Behar-Zusman et al., 2020; Zhang, 2022; Zilanawala et al., 2020). Second, a greater number of people in the household may have resulted in less personal space while physical distancing measures were in place, which could add to individuals’ own struggles with maintaining mental well-being.

Third, individuals with more housemates during the pandemic had more people’s well-being and health to consider. This may have created additional pressure to be careful not to bring the virus into the house, and it may have also created a sense of responsibility to help maintain the mental and emotional well-being of others. Those who were able to provide care would have faced greater demand for care from household members who were now staying home for extended periods. The caregiving burden may be heaviest among the (able-bodied) older adults in the household, especially as the middle generation is often not available due to HIV mortality (Schatz & Seeley, 2015). This caregiving demand may have caused heightened stress and anxiety for the older generation within households, and may have been especially pronounced for women, who were more likely to take on care and emotional work (Farré et al., 2022; Harriman et al., 2024).

#### Age composition

The composition of household members likely has important implications, beyond just the number of members (Punch, 2001). A key aspect of the mechanisms that we expect to impact anxiety is the number of dependents in a household, which can be approximated by the age composition of the household. Children and older household members are more likely to require care, as compared with young and middle-aged adults. Adults—especially women—living with a greater number of children or older adults may have faced an exacerbated risk of stress and anxiety due to caregiving demands while physically distancing.

Co-residing with a greater number of children or older adults may have also increased individuals’ anxiety during the early phase of the pandemic due to a heightened sense of responsibility to avoid bringing the COVID-19 virus into the home. In the early days of the pandemic, people had a strong motivation to protect children and older adults from the virus, as both were considered to be potentially vulnerable populations at the time (although children were later found to generally face a low risk of complications from the virus) (Girdhar et al., 2020; Kuy et al., 2020; Siebach et al., 2021). These unique concerns over household members’ health and well-being may have led physical distancing to further increase anxiety.

#### Gender composition

Gender composition of the household may have also been important, and even sometimes protective against the detriments of physical distancing on anxiety. Living in a household with more women may buffer against the impacts of physical distancing on anxiety for men and women alike because it means that more women are available to assist in or perform the care and emotional work (Settles et al., 2009). This relieves the burden on both men and other women in the household, but likely has an especially strong impact on other women who can then share the caregiving burden (vs men who may not have taken on this burden either way).

Women living with more women may also benefit from having more social interaction with same-gender housemates (Bedrov & Gable, 2023). Likewise, men living with more men may have benefited from greater social connection and bondedness amongst them. However, it is possible that households with more men increased caregiving burden for coresident women (Schatz & Seeley, 2015), thereby increasing stress and anxiety. Thus, living with more men has the potential to exacerbate the association between physical distancing and anxiety for women and mitigate the association for men.

### The role of gender in how household composition may impact anxiety

The gender of the focal individual is inextricably linked to how these processes may play out. Studies have shown that women fared worse in their mental health during the early months of the pandemic, compared with men (Etheridge & Spantig, 2020; Wilson-Genderson et al., 2022). This may be due to a number of factors, including greater loneliness felt by women than men during the pandemic (Etheridge & Spantig, 2020), stronger responses to stress and change (Silva et al., 2022), and a heavier caregiving burden (Zamarro & Prados, 2021). Previous studies have documented the detrimental effect that women’s caregiving and concerns over the emotional well-being of their household members had on their own mental health (Taniguchi et al., 2022; Zamarro & Prados, 2021). At the same time, the societal expectation for men to assume financial responsibility for their households may also make them particularly susceptible to anxiety while physically distancing, especially if it meant a disruption to their work and income (Lee et al., 2021). For these reasons, our empirical analyses consider the gender of the focal individuals at the forefront in informing our expectations, and we stratify our investigation by gender.

## Method

### Data and sample

We use data from the HAALSI study that follows a cohort of aging Black South African adults in Agincourt, South Africa. Participants were sampled from the existing framework of the Agincourt Health and Socio-Demographic Surveillance System (Agincourt HDSS) site in Mpumalanga province. Individuals 40 years and older as of July 1, 2014, and permanently living in the study site during the 12 months before the 2013 Agincourt census update were eligible. From these data, a sampling frame of 12,875 was identified and, using gender-specific sampling fractions to ensure a gender-balanced cohort, 6,281 were randomly selected to participate. A total of 5,059 individuals completed a wave 1 interview in 2014/2015, conducted using computer-assisted personal interviewing (CAPI) in the local language of Shangaan. The following two waves of the CAPI survey sampled all the living members of this original 5,059 HAALSI cohort. Wave 2 was conducted between October 2018 and November 2019, with a response rate of 94%. Wave 3 was conducted between July 2021 and March 2022, also with a response rate of 94%.

A COVID-19 phone survey was fielded from July 2021 to March 2022, overlapping with the timing of fieldwork for wave 3. This survey used computer-assisted telephone interviews (CATI) to gather information about the experiences of this cohort during the COVID-19 pandemic, hereafter referred to as the COVID-19 Survey. The COVID-19 Survey sampled all living members of the original 5,059 HAALSI cohort as of June 2021 (*n* = 4,247); *n* = 309 of these sampled individuals were found to have died upon contact attempt (Harriman et al., 2024). The survey concluded with a response rate of 69%. While modest, this response rate is comparable to other phone interviews during the COVID-19 pandemic (Collinge & Bath, 2023; Farina & Ailshire, 2022). Comparing with the full HAALSI sample, younger members of the sampled cohort were more likely to participate, as were women, those without limitations in their activities of daily living (ADLs), those with lower depression scores on the Center for Epidemiologic Studies Depression (CES-D), those with higher cognition, and those with stronger support networks. Our results will, therefore, reflect a younger, healthier, more socially connected, and higher proportion female sample than the larger HAALSI cohort.

Our analytic sample includes all members of the original HAALSI cohort who completed the COVID-19 Survey. Respondents who were missing information on measures from the COVID-19 Survey (*n* = 42) or measures from the HAALSI main surveys (*n* = 452) were excluded. Across all variables included in this analysis, the percent missingness of each variable ranged from 0% to 6.9%, and about 12% are only missing information on one variable. Hence, missing information was dispersed across variables and is not systematically correlated with the outcome (anxiety) or with the association between our key measures. Our analytic sample consists of 1,290 women and 914 men across 2,054 households. Data can be accessed via links found here: https://haalsi.org/haalsa-data/.

### Measures

#### Dependent variable

Our primary outcome measure is a modified version of the 2-item Generalized Anxiety Disorder scale (GAD-2), a screening tool used in other studies to indicate anxiety in a person by asking two questions related to their anxiety and worry over the past 2 weeks. This modified GAD-2 comes from the COVID-19 Survey and was used to assess an increase in anxiety during the pandemic. The questions asked were (a) “Since March 2020, relative to before the pandemic, have you felt nervous, anxious, or on edge more often, less often, or about the same?” and (b) “Since March 2020, relative to before the pandemic, how often have you felt you were not able to stop or control worrying? Would you say more often, less often, or about the same?” We coded responses on each measure as −1 (less often), 0 (about the same), and 1 (more often). We then averaged and dichotomized the averaged measure, such that a positive value was coded as 1, indicating that anxiety/worry increased, and values of 0 or less were coded as 0, indicating that anxiety/worry decreased or stayed the same. We used this cut-off threshold to facilitate interpretation and to align with past work that has used this same outcome measure (Harriman et al., 2024). Reliability tests for these measures indicate good internal consistency (Harriman et al., 2024).

#### Independent variable

The primary predictor measure in our analysis indicates a decline in extra-household in-person social contact. This measure was coded from a series of items in the COVID-19 Survey. First, respondents were asked, “Has the amount of in-person contact with your children living outside the household increased, decreased, or remained about the same since the COVID-19 pandemic began, relative to before the pandemic?” Next, respondents were asked the same about grandchildren, other family members who lived outside the household, and friends and neighbors (friends and neighbors combined as one group). We coded responses on each item as −1 (increased), 0 (remained about the same), and 1 (decreased). Respondents who did not have children or grandchildren were coded as missing on those items. Responses were then averaged and dichotomized, so that positive values were coded as 1, indicating that contact decreased, and values of zero or less were coded as 0, indicating that contact increased or remained the same. We used this coding scheme to facilitate interpretability and to align with past work (Harriman et al., 2024).

#### Modifying variables

We assess the modifying role of household size, as measured by the number of household members. We also assess the modifying role of the number of women in the household, the number of men in the household, the number of older adults (ages 60+) in the household, and the number of children (under age 15) in the household. These measures come from the wave 2 survey. The measures indicating the number of women and the number of men in the household were each coded to exclude the respondent from these counts. Additionally, for these measures, only household members who were between 15 and 60 years old were included, in order to create mutual exclusivity with the number of children and number of older adults, and to account more accurately for household members who are not of dependent ages (Madhavan et al., 2009). The measure of the number of older adult household members was also coded to exclude the respondent from the count, and was further dichotomized to none versus one or more older adult household members due to small cell sizes. Measures of the number of permanent household members and the number of men, women, and children were treated as continuous variables.

#### Covariates

We control for other measures that may also be associated with anxiety. In models estimating main effects, we account for a measure of the number of household members, coded as above. We adjust for age at wave 2, treated as a continuous measure. We also adjust for education as reported at the baseline (wave 1) interview, categorized as (a) no formal education, (b) some primary education (1 to 7 years of school), (c) some secondary education (8 to 11 years of school), and (d) completed secondary education or higher (12 or more years of education). We account for employment status at wave 2, coded as 1 if the respondent identified their primary employment status as employed or homemaker (translated to be understood as “one who manages the home”) and 0 if they identified as not employed or retired.

We adjust for marital status at wave 2, categorized as (a) currently married or living with a partner, (b) never married, (c) separated or divorced, or (d) widowed. We also account for the number of children respondents had at wave 1, with a continuous measure. We account for household wealth quintiles at wave 2. Households were ranked according to the scores from principal components analysis of household ownership of items such as televisions, refrigerators, livestock, vehicles, as well as housing characteristics, type of water and sanitation facilities (Riumallo-Herl et al., 2019). The Cronbach’s alpha for wealth quintiles is 0.66.

We also account for limitations in ADLs at wave 2, coded as 1 if the respondent had at least one ADL limitation and 0 if they had none. We account for comorbidities at wave 2, categorized as having either no comorbidities or at least one comorbidity (HIV, diabetes, and/or hypertension).

We also adjust for the month that the respondent completed the COVID-19 Survey, from July 2021 through March 2022. Due to small cell sizes, November 2021 through March 2022 were combined into a single category. We also account for mental health using a continuous measure of the 20-item CES-D scale at wave 2 (ranging from 0 to 60). We further control for a binary measure of wave 3 vaccination status, coded as 1 if vaccinated against COVID-19 and 0 if not vaccinated.

Next, we account for a binary measure indicating whether the respondent is the head of the household, which comes from the most recent Agincourt HDSS census data update as of 2021. Head of household is defined as the person who leads and makes household decisions, which is usually the oldest male, if a male is present. Finally, we adjust for a binary measure to indicate whether the respondent reports that they felt like they had the necessary help with household chores. This measure comes from the COVID-19 Survey.

### Analytic plan

To account for the potential for multiple participants to reside in the same household, generalized estimating equations (GEE), with a logit link, were used for these analyses. Household ID is treated as the clustering variable. Listwise deletion was used for missing data. Model specification was assessed by the quasi-likelihood information criterion. Because the role of household composition in the associations we investigate is inextricably linked with gender, we stratify analyses by gender. Separate models were assessed for effect modification of the number of people, the number of women, the number of men, the number of older adults, and the number of children living in the household. All analyses were performed using Stata 17.0. Because we use GEE with a logit link, results are presented as odds ratios.

## Results

Descriptive characteristics are shown in Table 1. Descriptive variables are summarized with means, standard deviations (*SD*s), medians, and ranges for continuous variables, and frequencies and proportions for categorical variables. For women, 36.1% reported an increase in anxiety symptoms since March 2020, compared with 28.9% among men. Among women, 62.9% reported a decrease in extra-household in-person social contact since March 2020, compared with 57.8% for men. Women were living in households with an average of 6.0 members (median of 5), an average of 1.5 male household members (median of 1), an average of 1.4 female household members (median of 1), and an average of 1.9 children (median of 2). Twenty-three percent of women lived with at least one older adult household member. Men were living in households with, on average, 5.6 members (median of 5), 1.2 male members (median of 1), 1.5 female members (median of 1), and 1.6 children (median of 1). Thirty-three percent of men lived with at least one older adult household member.

**Table 1:** Sample characteristics.

Variables	Women (*n *= 1,290)		Men (*n *= 914)	
	Proportion (%)/mean (*SD*)	Median (range)	Proportion (%)/mean (*SD*) %	Median (range)
**Dependent measure**				
Increase in anxiety symptoms since March 2020	36.1		28.9	
**Independent measure**				
Decline in in-person social interactions since March 2020	62.9		57.8	
**Potential modifiers**				
Number of permanent household members	6.0 (3.5)	5 (1–26)	5.6 (3.6)	5 (1–24)
Number of male household members^a^	1.5 (1.4)	1 (0–8)	1.2 (1.3)	1 (0–10)
Number of female household members^a^	1.4 (1.4)	1 (0–10)	1.5 (1.4)	1 (0–9)
Number children household members	1.9 (1.8)	2 (0–10)	1.6 (1.8)	1 (0–11)
One or more older adult household members	23.5		33.5	
**Covariates^b^**				
**Age**	63.2 (11.4)	62 (43–100)	63.9 (11.3)	64 (44–100)
**Education**				
No formal education	45.2		34.7	
Some or complete primary (1–7 years)	35.8		39.6	
Some secondary (8–11 years)	11.0		15.8	
Secondary or more (12+ years)	8.0		10.0	
**Work status**				
Not working/retired	84.5		77.0	
Employed or home manager	15.5		23.0	
**Marital status**				
Never married	4.9		9.1	
Separated or divorced	11.9		11.7	
Widowed	47.7		10.9	
Currently married or living with partner	35.5		68.3	
**Number of children <15 years old**	4.5 (2.2)	5 (0–8)	4.7 (2.5)	5 (0–8)
**Wealth asset index quintiles**				
Q1—Poorest	18.8		19.5	
Q2—Poor	18.4		17.6	
Q3—Middle	18.5		19.8	
Q4—Less poor	20.4		21.6	
Q5—Least poor	24.0		21.6	
**Has at least one ADL limitation**	5.0		5.6	
**Has at least one comorbidity**	79.3		70.9	
**Month COVID-19 survey was completed**				
July 2021	7.1		7.9	
August 2021	20.6		20.7	
September 2021	27.1		27.8	
October 2021	37.4		36.2	
November 2021–March 2022	7.8		7.4	
**CES-D score**	14.7 (9.3)	13 (0–51)	13.5 (9.4)	12 (0–43)
**Vaccinated for COVID-19**	59.2		57.1	
**Respondent is the head of the household**	57.3		92.0	
**Felt like they had the necessary help with household chores**	80.2		78.6	

*Note*. *SD* = standard deviation; Q = quintile; ADL = activities of daily living; CES-D = Center for Epidemiologic Studies Depression Scale.

a Between the ages of 15 and 60 years.

b All covariates measured at wave 2 except education from wave 1; vaccination status from wave 3; “Month COVID-19 survey completed” and “felt like they had the necessary help with household chores” from the COVID-19 Survey; and “respondent is the head of the household” from the Agincourt census (HDSS).

In Table 2, we investigate the associations between the decline in in-person social interactions since the start of the pandemic and anxiety for women. We find that a decline in in-person interactions was associated with greater odds of increased anxiety (OR = 2.142, *p* < 0.001) among women.

**Table 2:** Generalized estimating equations predicting increase in anxiety, women (*N* = 1,290).

Variables	OR	*SE*	*p*
**Independent variable**			
Decline in in-person social contact since March 2020	2.142	0.285	<.001
**Covariates**			
Number of permanent household members	1.031	0.019	.101
**Age**	0.999	0.00683	.837
**Education**			
No formal education		*Ref*	
Some primary education (1–7 years)	1.124	0.161	.413
Some secondary education (8–11 years)	1.128	0.245	.579
Secondary education or more (12+ years)	1.331	0.352	.279
**Work status**			
Not working/retired		*Ref*	
Employed or home manager	1.303	0.228	.130
**Marital status**			
Currently married or living with a partner		*Ref*	
Never married	0.603	0.191	.111
Separated or divorced	0.716	0.173	.165
Widowed	0.598	0.122	.012
**Number of children <15 years old**	0.978	0.031	.476
**Wealth asset index quintiles**			
Wealth Index Q1—poorest		*Ref*	
Wealth Index Q2—poor	0.867	0.176	.483
Wealth Index Q3—middle	0.982	0.198	.927
Wealth Index Q4—less poor	1.073	0.212	.722
Wealth Index Q5—least poor	0.896	0.179	.582
**Has at least one ADL limitation**	0.778	0.233	.402
**COVID-19 questionnaire month**			
July 2021		*Ref*	
August 2021	1.532	0.439	.136
September 2021	1.106	0.311	.721
October 2021	1.511	0.414	.132
November 2021–March 2022	2.906	0.969	.001
**CES-D score**	1.015	0.007	.026
**Has at least one comorbidity**	1.153	0.177	.355
**Vaccinated for COVID-19**	0.989	0.126	.930
**Respondent is the head of the household**	1.401	0.258	.067
**Felt like they had the necessary help with household chores**	0.490	0.084	<.001

*Note.* OR = odds ratio; *SE* = standard error; ADL = activities of daily living; CES-D = Center for Epidemiologic Studies Depression Scale.

**Figure 1:**
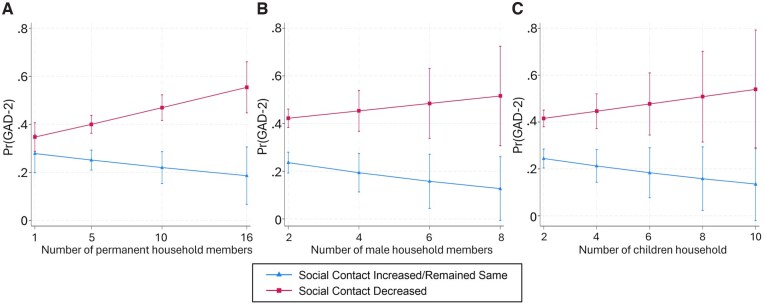
Association between physical distancing and anxiety by modifying roles: (A) number of household members; (B) number of male household members; and (C) number of children household members.

In Table 3, we investigate whether household characteristics modify the association identified in Table 2. All covariates from models in Table 2 are included here as well, though not shown for brevity. We find that living in larger households was a significant modifier of this association: women living in larger households experienced a larger positive association between decline in in-person interaction and odds of increased anxiety (OR = 1.096, *p* = 0.026; Model 1). We also find that the number of men in the household was a significant modifier (OR = 1.201, *p* = 0.065, Model 2), as was the number of children in the household (OR = 1.165, *p* = 0.049; Model 4). Living with more men or more children increased the magnitude of the association between a decline in in-person interaction and the odds of increased anxiety for women. These significant modifying effects are displayed in Figure 1. We find null results for the number of women and the number of older adults in the household as possible modifiers among women (Models 3 and 6, respectively).

**Table 3:** Generalized estimating equations predicting an increase in anxiety and the modifying role of household composition, women (*n *= 1,290).

Variables	Model 1: Modification by household size			Model 2: Modification by number of men in household			Model 3: Modification by number of women in household			**Model 4: Modification by number of children in household**			Model 5: Modification by number of older adults in household		
	OR	*SE*	*p*	OR	*SE*	*p*	OR	*SE*	*p*	OR	*SE*	*p*	OR	*SE*	*p*
**Decline in in-person social contact since March 2020**	1.257	0.339	.397	1.624	0.319	.014	1.780	0.337	.002	1.619	0.313	.013	2.077	0.313	<.001
**Number of permanent household members**	0.966	0.036	.349	1.031	0.023	.170	1.041	0.032	.193	1.022	0.032	.482	1.031	0.019	.109
** x Decline in in-person social contact since March 2020**	1.096	0.045	.026												
**Number of male household members**				0.882	0.081	.171									
** x Decline in in-person social contact since March 2020**				1.201	0.123	.065									
**Number of female household members**							0.874	0.094	.210						
** x Decline in in-person social contact since March 2020**							1.153	0.119	.116						
**Number of children household members**										0.913	0.074	.265			
** x Decline in in-person social contact since March 2020**										1.165	0.091	.049			
**One or more older adult household members**													0.934	0.273	.816
** x Decline in in-person social contact since March 2020**													1.141	0.363	.679

*Note*. OR = odds ratio; *SE* = standard error. All covariates in Table 2 are included here but not shown.

In Tables 4 and 5, we investigate these same associations among men. Again, all covariates from models in Table 2 are included here, though not shown. In Model 1 of Table 4, we find that a decline in in-person interactions was associated with greater odds of increased anxiety (OR = 2.722, *p* < 0.001) among men, similar to women.

**Table 4:** Generalized estimating equations predicting an increase in anxiety, men (*n *= 914).

Variables	OR	*SE*	*p*
**Independent variable**			
Decline in in-person social contact since March 2020	2.722	0.457	<.001
**Covariates**			
Number of permanent household members	1.008	0.024	.735
**Age**	0.991	0.009	.334
**Education**			
No formal education		*Ref*	
Some primary education (1–7 years)	1.106	0.206	.589
Some secondary education (8–11 years)	0.704	0.190	.193
Secondary education or more	1.305	0.409	.396
**Work status**			
Not employed or retired		*Ref*	
Employed or home manager	0.906	0.186	.632
**Marital status**			
Currently married or living with a partner		*Ref*	
Never married	1.582	0.443	.102
Separated or divorced	1.178	0.324	.551
Widowed	0.796	0.214	.397
Number of children <15 years old	0.985	0.038	.692
**Wealth asset index quintiles**			
Wealth Index Q1—poorest		*Ref*	
Wealth Index Q2—poor	1.099	0.291	.722
Wealth Index Q3—middle	1.253	0.321	.379
Wealth Index Q4—less poor	1.297	0.331	.308
Wealth Index Q5—least poor	1.115	0.295	.681
**Has at least one ADL limitation**	2.377	0.794	.010
**COVID-19 questionnaire month**			
July 2021		*Ref*	
August 2021	1.091	0.352	.788
September 2021	1.091	0.343	.782
October 2021	1.063	0.324	.840
November 2021–March 2022	1.799	0.698	.130
**CES-D score**	0.996	0.009	.664
**Has at least one comorbidity**	1.644	0.295	.006
**Vaccinated for COVID-19**	0.998	0.158	.990
**Respondent is the head of the household**	1.485	0.471	.212
**Felt like they had the necessary help with household chores**	0.553	0.114	.004

*Note.* OR = odds ratio; *SE* = standard error; ADL = activities of daily living; CES-D = Center for Epidemiologic Studies Depression Scale. All covariates in Table 2 are included here but not shown.

**Table 5:** Generalized estimating equations predicting an increase in anxiety and the modifying role of household composition, men (*n *= 914).

Variables	Model 1: Modification by household size			Model 2: Modification by number of men in household			Model 3: Modification by number of women in household			**Model 4: Modification by number of children in household**			Model 5: Modification by number of older adults in household		
	OR	*SE*	*p*	OR	*SE*	*p*	OR	*SE*	*p*	OR	*SE*	*p*	OR	*SE*	*p*
**Decline in in-person social contact since March 2020**	2.696	0.804	<.001	2.886	0.680	<.001	2.590	0.606	<.001	2.572	0.565	<.001	3.241	0.688	<.001
**Number of permanent household members**	1.007	0.039	.857	0.974	0.029	.379	1.019	0.038	.603	1.073	0.045	.093	1.002	0.024	.941
** x Decline in in-person social contact since March 2020**	1.002	0.044	.969												
**Number of male household members**				1.182	0.144	.170									
**x Decline in in-person social contact since March 2020**				0.960	0.123	.751									
**Number of female household members**							0.940	0.113	.607						
**x Decline in in-person social contact since March 2020**							1.036	0.117	.754						
**Number of children household members**										0.840	0.090	.102			
**x Decline in in-person social contact since March 2020**										1.033	0.099	.736			
**One or more older adult household members**													1.855	0.581	.048
**x Decline in in-person social contact since March 2020**													0.593	0.207	.135

*Note*. OR = odds ratio; *SE* = standard error. All covariates in Table 2 are included here but not shown.

In Table 5, we investigate the modifying role of different household characteristics. As above, all covariates from models in Table 2 are included here, but not shown. In Models 1 through 6, we find null associations for modifiers of household size, number of men in the household, number of women in the household, number of children in the household, and number of older adults in the household, respectively.

Sensitivity analyses were performed to test whether the age cut-offs used to define older adults and children are robust. These analyses showed that using an age cut-off of 75 years for older adults and 12 years for children did not substantively change the results from those shown here.

## Discussion

We investigated the role of household composition in how social isolation may impact anxiety among an aging cohort in rural South Africa. Data collected during the COVID-19 pandemic gives us unique insight into the consequences of social isolation that was imposed by public health mandates. We found that both women and men who reported a decline in in-person interactions were more likely to experience an increase in anxiety symptoms during this period. However, only among women did we find significant evidence that household composition may impact these associations: women who were living in larger households, in households with more men, or in households with a greater number of children had greater odds of increased anxiety in response to a decline in in-person social interaction.

Our results offer evidence that the anxiety of both women and men was negatively impacted by extra-household physical distancing in this rural South African setting, where households tend to be large. While we expected that physical distancing may have been less harmful to the mental health of older adults in such a setting, given the greater opportunity for social interaction with coresident family, our findings mirrored those in other settings that found people’s anxiety to have increased in the early months of the COVID-19 pandemic (Benke et al., 2020; Chen et al., 2021). There was also reason to expect that men may not be as impacted as women, but our results indicate that both women and men reported increased anxiety in response to physical distancing. Men may have experienced increased anxiety, especially due to stress related to disruptions in work and income (Lee et al., 2021), while women may have been especially impacted by the loss of caregiving support from outside of the home (Taniguchi et al., 2022; Zamarro & Prados, 2021). Our subsequent analyses of the moderating impacts of household composition offered more insight into the gender-specific factors that might have exacerbated anxiety.

We found that the aging women in our sample who were living in larger households or in households with a greater number of men or children reported a greater increase in anxiety symptoms in association with physical distancing. The moderating impacts of these household composition indicators were not found to be significant for men. Women may have dealt with more stress if they lived in larger households, or if they lived with a greater number of men or children. Across settings, women more often take on caregiving and emotional support tasks than men (Revenson et al., 2016), and may take on this role even more disproportionately in this rural South African setting (Schatz & Seeley, 2015). Older South African women tended to take on the role of head of household when Apartheid-era labor and housing policies resulted in men leaving their homes for work (Hosegood et al., 2009). Although these policies have since ended, they have had a lasting impact on labor migration and family composition (Budlender & Lund, 2011; Smit, 2001), continuing to leave many households headed by women. Moreover, the middle generation faced high mortality due to HIV, and older women—such as those in our sample—often fill these caregiving gaps for their grandchildren (Schatz & Seeley, 2015). These realities, combined with the pervasiveness of masculine social norms, may leave the aging women in our sample to assume a great deal of caregiving burden when living with more people, in general, and especially with more children or more men.

The caregiving burden would have increased during the lockdowns and early stages of the pandemic, as household members requiring care would have been staying home much more than before. Caregivers likely increased their work around the home, including cooking, cleaning, and looking after the well-being of household members (Farré et al., 2022). This increase in caregiving burden coincided with a decline in access to social support from outside of the home. Caregivers were faced with a simultaneous increase in demands on their caregiving and a decrease in access to support, such as emotional and instrumental support, that could have helped them to manage the burden (Harriman et al., 2024). These factors shed further light on the possible reasons for our gender-specific results regarding the modifying role of household size, number of coresident children, and number of coresident men.

We did not find indicators of household size, age, or gender composition to be significant modifiers in the association between physical distancing and increased anxiety for men. We also did not find that women living with older adults experienced exacerbated impacts of physical distancing, nor that women living with more women were protected against the negative impacts, as we expected. Children may have had a greater demand for caregiving during the pandemic than older adults, which could help to explain why we found the former but not the latter to significantly modify the association. Regarding the null results for the number of coresident women, this may be because the caregiving burden primarily falls on one woman in the household—usually the grandmother (Cantillon et al., 2021)—so that additional women did not alleviate the burden. Combined, our findings on household gender composition offer an important contribution to knowledge of how these dynamics may matter for home-bound adults: coresident women may neither protect against nor exacerbate the association between physical distancing and anxiety, but a greater number of coresident men may be an added detriment in this association for women.

Taken together, these findings have important implications, adding to the extensive literature that highlights the negative impacts of social isolation on mental health (Benke et al., 2020; Leigh-Hunt et al., 2017; Rohde et al., 2016; Xiao et al., 2020). Our findings extend this work to the context of LMICs, suggesting that social isolation, in the form of physical distancing during a pandemic, may have similar detrimental impacts among this aging cohort of men and women in South Africa. Despite the potential for larger households to facilitate greater access to social interaction and support from within, we find evidence among our sample of women that living in larger households, in fact, exacerbates the negative impacts of extra-household physical distancing. It is therefore important for interventions targeted at socially isolated groups to build support systems that can offset some of the anxiety-inducing aspects of social isolation. Successful interventions might include offering greater guidance and encouragement for individuals to safely interact across households, including remote (or virtual) forms of social interaction, which have been found to mitigate some of the negative associations between physical distancing and anxiety (Harriman et al., 2024).

Our investigation had a number of limitations to note. First, our key independent and dependent measures were retrospectively self-reported. The accuracy of respondents’ recall of anxiety and in-person interactions over the past 16–24 months may be limited. Nonetheless, these self-reported measures offer unique insight into how respondents perceived their situation to change during the pandemic. Second, the COVID-19 Survey had a relatively low response rate, compared with surveys conducted in-person among this cohort. Telephone interviews tend to produce lower response rates across settings (Groves & Couper, 1998). These lower response rates can result in selectivity of our sample, and may bias our results and limit generalizability. Finally, results from this Agincourt sample and from data collected during the COVID-19 pandemic are further limited in their generalizability due to specific characteristics of the study site. While we expect relevance for other rural SSA communities (Collinson et al., 2022), and for social isolation during non-pandemic times, further research is needed to understand generalizability beyond this setting and time period.

Overall, our study offers important insight into the impact of social isolation on anxiety outcomes and how this impact is modified by the demographics of one’s household. We found that, while both women and men who experienced a decline in in-person interactions during the COVID-19 pandemic were more likely to experience an increase in self-reported anxiety symptoms, this association was made worse for women living in larger households, households with more men, or households with a greater number of children. We speculate that these gender-specific findings are largely due to social norms that lead women to assume most caregiving responsibilities, combined with increased caregiving demands and decreased access to support in fulfilling them. Our results can inform the development of policies and interventions designed to ameliorate population mental health and redress gender health inequities in future public health emergencies and among groups most at risk of social isolation.

## Data Availability

The data underlying this article are available in Harvard Dataverse at https://doi.org/10.7910/DVN/Q2JFPV and https://doi.org/10.7910/DVN/DDNWFA, and at the Inter-university Consortium for Political and Social Research (ICPSR) at https://doi.org/10.3886/ICPSR36633.v4.
